# Necrology

**Published:** 1900-08

**Authors:** 


### NECROLOGY.

JOHN A. PEARSON, V.M.D. At Bryn Mawr, Pa., August 4, 1900, Dr. Pearson, a graduate of the Veterinary Department of the University of Pennsylvania, met a sad and distressing death.

While endeavoring to control and teach an unmanageable horse to be fearless of steam he was thrown down an embankment from his cart and the horse followed, rolling upon him, injuring him so badly that he lived but a few hours.

Dr. Pearson was formerly a resident and later a practitioner of Kansas. For several years he had been in Philadelphia, where he gave the most of his time to handling and training horses for the saddle and driving. He was but thirty-three years old, and leaves a wife.

